# Comparative Study of Mesenchymal Stem Cell Identity and Chondrogenic Differentiation Capacity of Human Nucleus Pulposus, Adipose and Bone Marrow Tissues Derived Mesenchymal Stem Cells

**DOI:** 10.1007/s13770-026-00824-2

**Published:** 2026-07-22

**Authors:** Hataiwan Kunkanjanawan, Tanut Kunkanjanawan, Veerapol Khemarangsan, Siravich Suvithayasiri, Tawit Suriyo, Jutamaad Satayavivad, Warayos Trathitephun

**Affiliations:** 1 Medeze Research and Development Co., Ltd., 28/9 Moo 8, Phutthamonthon Sai 4 Rd., Krathum Lom, Sam Phran, Nakhon Pathom 73220 Thailand; 2 Medeze Group Pub. Co., Ltd., 28/9 Moo 8, Phutthamonthon Sai 4 Rd., Krathum Lom, Sam Phran, Nakhon Pathom 73220 Thailand; 3 Orthopedics Department, Chulabhorn Hospital, 906 Kamphaeng Phet 6 Rd, Talat Bang Khen, Lak Si, Bangkok 10210 Thailand; 4 Laboratory of Pharmacology, Chulabhorn Research Institute (CRI), 54 Kamphaeng Phet 6 Rd, Khwaeng Talat Bang Khen, Khet Lak Si, Bangkok 10210 Thailand; 5 Center of Excellence on Environmental Health and Toxicology (EHT), Ministry of Higher Education, Science, Research and Innovation (MHESI), OPS, Bangkok, Thailand

**Keywords:** Mesenchymal stromal cells, Bone marrow, Adipose tissue, Nucleus pulposus, Chondrogenic differentiation, IVDD

## Abstract

*****BACKGROUND***:**

Mesenchymal stem cells (MSCs) are multipotent mesoderm-derived cells with high proliferation capacity, self-renewal, paracrine activity, and multilineage differentiation potential. Autologous MSCs from the same patients are particularly valuable for regenerative therapies, including those targeting degenerative intervertebral disc disorder (DIVDD).

*****METHODS***:**

In this study, we directly compared autologous MSC sources obtained from the same patients, thereby reducing biological variability and strengthening translational relevance. We compared MSCs isolated from bone marrow (BM–MSCs), adipose tissue (AD–MSCs), and nucleus pulposus (NP–MSCs) obtained from the same DIVDD patient donor (*N* = 10 donors). We assessed their morphologies, proliferation abilities, immunophenotype, multilineage differentiation capacity, and chondrogenic differentiation–related gene and protein expression.

*****RESULTS***:**

All three MSC populations displayed similar fibroblast-like morphology and consistent immunophenotypic profiles. However, their functional properties differed: AD–MSCs exhibited significantly higher proliferative activity and greater adipogenic differentiation potential (*p* < 0.01 and *p* < 0.001), while osteogenic and chondrogenic differentiation capacities were comparable across BM-MSCs, AD-MSCs, and NP-MSCs at both gene and protein levels (SOX9 and COMP).

*****CONCLUSION***:**

These findings suggest that AD-MSCs offer practical advantages in terms of proliferation capacity and accessibility. However, chondrogenic differentiation capacity was largely comparable across MSC sources. Further mechanistic studies and *in vivo* validation are required to determine their relative therapeutic efficacy for intervertebral disc regeneration.

## Introduction

The intervertebral disc (IVD) is a specialized cartilage-like connective tissue located between adjacent vertebral bodies, functioning to absorb mechanical load and maintain multiaxial spinal flexibility. The nucleus pulposus (NP), a gelatinous core surrounded by the annulus fibrosus (AF) and thin hyaline cartilaginous endplates (CEP), represents a unique avascular niche characterized by hypoxia, acidity, low nutrient supply, and sparse cellularity [1]. NP cells (NPCs) play a central role in extracellular matrix (ECM) production and disc homeostasis. In degenerated intervertebral disc disorder (DIVDD), age, mechanical stress, and reduced osmotic pressure contribute to progressive ECM loss and declining NPC function. Consequently, cell density decreases and intrinsic repair mechanisms become insufficient, leading to impaired regeneration [2].

Conventional treatments for DIVDD, including physical therapy, pain management, and surgery, primarily addressed symptoms rather than restoring disc structure or function. These approaches often result in persistent pain, adjacent segment disease, or revision surgery [3]. Stem cell-based strategies, particularly those employing mesenchymal stem cells (MSCs), have therefore emerged as promising alternatives for disc preservation and regeneration.

MSCs are multipotent, mesoderm–derived stem cells isolated from a variety of tissues, including bone marrow, adipose tissue, umbilical cord, placenta, and NP itself [2–4]. They share core biological features such as plastic adherence, characteristic surface markers, high proliferative potential, and multilineage differentiation capacity, making them attractive for clinical applications [5]. Among these, bone marrow-derived MSC (BM-MSCs), adipose-derived MSC (AD-MSCs), and NP-derived MSC (NP-MSCs) from DIVDD patients have gained particular interest due to their low immunogenicity, autologous applicability, and relevance to cartilage-like tissue repair [2, 3]. However, reports on their chondrogenic potential remain inconsistent [2, 3, 6, 7]. While NP-MSCs resemble native cartilage cells, their regenerative capacity is often compromised by donor age and degeneration severity [8]. Some studies suggest that AD-MSCs exhibit comparable or superior chondrogenic capacity to BM-MSCs [2, 6], whereas others identify BM-MSCs as more favorable [9]. Similarly, comparative analyses of BM-MSCs with IVD-derived MSCs (NP-, AF-, and CEP-MSCs) have produced conflicting outcomes [7]. Importantly, transplantation studies in animal models and clinical trials indicate that all three MSC sources contribute to disc repair, albeit with variable outcomes [8, 10–12].

Given these discrepancies, a direct comparison of BM-, AD-, and NP-MSCs obtained from the same DIVDD patients is needed to reduce donor variability and clarify their relative therapeutic potential. Therefore, this study aimed to systematically evaluate the morphology, proliferation, immunophenotype, multilineage differentiation, and chondrogenic gene/protein expression of BM-, AD-, and NP-MSCs derived from the same patient donors. This work is among the few studies to directly compare autologous MSC sources obtained from the same patients, thereby minimizing donor-related variability and enhancing translational relevance.

## Materials and methods

### Human articular cartilage preparation

This study was approved by the Human Research Ethics Committee of Chulabhorn Royal Academy, Bangkok, Thailand. Human articular cartilage (*N* = 2) was obtained from a patient undergoing total knee replacement for osteoarthritis at the Chulabhorn Hospital, Bangkok, Thailand, after the patient had signed the informed consent. Human articular cartilage was used as a positive control in the experiments.

### Isolation and cultivation of human mesenchymal stem cells (hMSCs)

Human MSCs derived from bone marrow (BM), adipose (AD), and nucleus pulposus (NP) tissues were procured from ten patients (*N* = 10) who underwent posterior discectomy surgery for lumbar degenerative disease. The average age of the patients was 63.7 years (range: 52–72 years). The patient details are summarized in Table 1. The study was approved by the Human Research Ethics Committee of Chulabhorn Royal Academy, Bangkok, Thailand. (Ethics number: EC053/2566; Approval date: 6 October 2023) After receiving the signed informed consents from the patients, the BM, AD, and NP tissues were collected and processed at MEDEZE Research and Development laboratory within 24 h after delivery. In all experiments, cells were maintained in a humidified atmosphere of 37 $^\circ$C and 5% CO_2_ incubator.

**Table 1:** Characteristics of the patients enrolled in the study

Case No	Age (years)	Sex	Symptoms	Diagnosis	Disc level	Pfirrmann grade
1 2 3 4 5 6 7 8 9 10	61 60 52 60 69 71 68 72 63 61	M M F M M M F F F F	BP–RP BP-RP BP-RP RP BP-RP BP-RP BP-RP BP-RP BP-RP BP	Lumbar disc herniation Lumbar disc herniation Lumbar stenosis Lumbar disc herniation Lumbar disc herniation Lumbar stenosis Lumbar stenosis Lumbar stenosis Lumbar stenosis Lumbar disc herniation	L4/5 L4/5 L5/S1 L4/5 L4/5 L4/5 L4/5 L4/5 L4/5 L4/5	III IV IV IV IV V V V IV III

M, male; F, female; BP, back pain; RP, radicular pain

MSCs were isolated from BM, AD, and NP tissues from the same patients, using an enzymatic digestion method procedure as previously described [10, 13, 14], with some modification.

Briefly, the collected BM, AD and NP tissues (∼ 1–4 g) were sterilized by ethanol and rinsed twice by phosphate-buffered saline (PBS, Apsalagen, Bangkok, Thailand) in a 50-mL conical tube (Corning, Corning, NY, USA). The tissues were then digested with an equal volume of 0.1% (w/v) collagenase type I (Merck Millipore, Billerica, MA, USA) in alpha modification of Eagle’s medium (α-MEM, Corning) for 45 min for AD tissue and for 120 min for BM and NP tissues, and the cells were isolated by centrifugation at 1,000 × g at room temperature for 10 min. The cells were cultured in a 6–well plate (Costar, Corning, NY, USA) in culture medium comprised of α-MEM supplemented with 10% (v/v) human serum, 2 mM L-glutamine (Corning) and 100 μg/ml streptomycin and 100 U/ml penicillin (Sigma-Aldrich, St Louis, MO, USA). After three days, the unattached cells were depleted by replacing the medium with fresh medium. The medium was subsequently changed twice a week.

At 90% confluence, the BM–MSCs, AD–MSCs, and NP–MSCs were subcultured by 1% (v/v) TrypLE Express (Invitrogen, Carlsbad, CA, USA) + 0.1% (v/v) ethylenediaminetetraacetic acid (EDTA) solution (Sigma-Aldrich) and used for subsequent studies. For future use, the BM–MSCs, AD–MSCs, and NP–MSCs were frozen in freezing medium consisting of culture medium plus 10% (v/v) dimethylsulfoxide (DMSO, Wak-Chemie Medical GmbH, Steinbach (Taunus), Germany) and 20% (v/v) human serum. The frozen aliquots were then stored in vapor-phase liquid nitrogen.

### Expansion potential of hMSCs from different sources

To compare expansion potential in each hMSC source, growth kinetics of BM–MSCs, AD–MSCs, and NP–MSCs (from passage 4 to passage 6) were quantified as described previously [15, 16], with some modifications. Briefly, BM–MSCs (*N* = 10), AD–MSCs (*N* = 10) and NP–MSCs (*N* = 10) were plated at 1 × 10^4^ cells/cm^2^ and cultured under their original condition for 72 h. Then, cells from each group were collected, stained with 0.4% (v/v) trypan blue (Invitrogen), and counted by Countess™ Automated Cell Counter (Invitrogen). Each condition was performed in duplicate. Population doubling (PD) and population doubling time (PDT) was calculated using the equations (1) PD = (log NH − log NI)/log 2 and (2) PDT = (CT × ln2)/ln (NH/NI), respectively. NH is the harvested cell number, NI is the initial cell number, and CT is the cell culture time (days).

### Measurement of cell proliferation capacity

To confirm the proliferation capacity of differentially BM–MSCs, AD–MSCs, and NP–MSCs (from passage 4 to passage 6, each hMSCs sources; *N* = 10), the Cell Counting Kit–8 (CCK–8, Dojindo Laboratories, Japan) were used as described previously [3], with some modification. Briefly, 10 μL of CCK–8 solution was added to each well of a 96-well plate (SPL life sciences, Gyeonggi-do, Korea) containing 5 × 10^3^ cells/well. After incubating the plate at 37 °C for 4 h, absorbance at 450 nm was measured using a microplate absorbance reader (Azure, Redmond, Microsoft Way, USA). Cell proliferation was tested on day 3. A blank 96-well plate was used for the zero setting. All experiments were performed twice.

### Flow cytometry analysis

The purities of BM–MSCs, AD–MSCs, and NP–MSCs at passage 3 (each hMSC source, *N* = 10) were taken from flow cytometry analysis. The following anti–human antibodies were used according to the manufacturer’s instructions. For hMSCs, cells were stained with anti-HLA-DR-Alexa Fluor® 488 (clone: L243, #307619), anti-CD34-PE (clone: 581, #343505), anti-CD45-FITC (clone: HI30, #304006), anti-CD73 (Ecto-5-nucleotidase)-APC (clone: AD2, #344006), anti-CD90 (Thy1)-PE/Dazzle™ 594 (clone: 5E10, #328134), and anti-CD105-PE (clone: 43A3, #323206). All antibodies were purchased from BioLegends (San Diego, California, U.S.A.). Corresponding isotype immunoglobulins (IgG) were used as negative control. Single-cell suspensions of 1 × 10^5^ cells were incubated with the appropriate concentration of antibodies for 20 min at room temperature. Then, cells were washed and resuspended in 500 μL PBS and analyzed using a CytoFLEX flow cytometer (Beckman Coulter, Brea, CA, USA). At least 10,000 events were acquired, and the results were analyzed using CytExpert software.

### Multipotency assays

BM–MSCs, AD–MSCs, and NP–MSCs were cultured at the final density of approximately 2 × 10^4^ cells/cm^2^ in a 6-well culture plate (Costar) coated with 0.1% (w/v) gelatin (Sigma).

BM–MSCs, AD–MSCs, and NP–MSCs were induced to osteogenic differentiation by culture in the osteogenic differentiation medium (catalog number: C-28013; PromoCell GmbH, Heidelberg, Baden-Wurttemberg, Germany). The medium was subsequently replaced every 3 days for 4 weeks. Calcium deposits from the cells were then visualized by Alizarin Red staining.

To induce adipogenic differentiation, BM–MSCs, AD–MSCs, and NP–MSCs were cultured in the adipogenic differentiation medium (catalog number: C-28016; Promo Cell GmbH). The medium was subsequently replaced every 3 days for 3 weeks. Cells were then stained with Oil Red O to observe oil droplets.

To induce chondrogenic differentiation, BM–MSCs, AD–MSCs, and NP–MSCs were cultured in the chondrogenic differentiation medium (catalog number: C-28012; Promo Cell GmbH). The medium was replaced every 3 days for 3–4 weeks. Glycosaminoglycan (GAG) production was assessed by Alcian blue 8 × staining.

### Reverse transcription-quantitative polymerase chain reaction (RT-qPCR) analysis

After 0, 21, and 28 days of induction, total RNA was isolated from the cells by total RNA extraction kit (iNtRON Biotechnology, Seongnam, Kyonggi-do, South Korea) according to the manufacturer’s instructions. Then, RNA was reverse-transcribed in the presence of oligo-dT primer for complementary DNA (cDNA) synthesis by Maxime™ RT PreMix (iNtRON Biotechnology). The expression of several genes was assessed by using CFX96 Touch Real-Time PCR Detection System (Bio-Rad, Hercules, CA, USA) and KAPA SYBR-Green PCR Master Mix (Applied Biosystems, Carlsbad, CA, USA). The primers used are shown in Table 2.

**Table 2:** List of primer sequences used in this study

Genes	Primer Sequence	Annealing Temp. (^o^C)	Size (bp)	References
*SOX9*	Forward 5′-GGCAAGCTCTGGAGACTTCTG-3′ Reverse 5′-CTGCAGCGCCTTGAAGATG-3′	62	207	[21]
*COL2A1*	Forward 5′-GAGACAGCATGACGCCGAG-3′ Reverse 5′-GCGGATGCTCTCAATCTGGT-3′	62	67	[21]
*COMP*	Forward 5′-CCCCAATGAAAAGGACAACTGC -3′ Reverse 5′- GTCCTTTTGGTCGTCGTTCTTC -3′	62	121	[22]
*β-ACTIN*	Forward 5′-CTTTGGGCTGCTCGCTATGA-3′ Reverse 5′-GCATGTTGATTTAACAAGCTGCT-3′	62	219	[23]

Melting curve analysis was undertaken to determine the specificity of the primers. Gene expression was normalized to the reference gene *β–ACTIN* and calculated as the relative expression compared to control cells. The qPCR analyses were performed twice.

### Western blot analysis

Total protein was extracted from the samples 3 weeks post-induction by using Pierce RIPA lysis buffer (Thermo Fisher Scientific, Waltham, MA, USA) and complete protease inhibitor cocktail (Roche Diagnostics, Basel, Switzerland). The total protein concentration was determined by using the Bradford assay. Twenty micrograms of the protein sample were diluted in 2 × Laemmli’s sample buffer (Bio-Rad, CA, US) heated for 5 min at 95 °C on a digital dry bath/block heater (Thermo Scientific), and separated by sodium dodecyl sulfate–polyacrylamide gel electrophoresis (SDS PAGE), using a mini-PROTEAN® TGX™ Precast gradient 10% gel (Bio-Rad, CA, US), followed by electro-transfer onto nitrocellulose membranes (Bio-Rad). Nitrocellulose membranes were blocked non-specific proteins with 5% (w/v) skim milk (HiMedia) in 1X (v/v) Tris-buffered saline solution (Sigma) or SuperBlock™ (TBS) Blocking buffer (Thermo Fisher Scientific). Then, nitrocellulose membranes were probed with the primary and secondary antibodies as follows in Table 3. Signals were detected using a fluorescence system or a chemiluminescence reagent (ECL, Millipore, Burlington, MA, USA) according to the manufacturer’s instructions. Images were acquired by a ChemiDoc™ Digital Imaging System with image analysis software (ImageLab software, version 6.0.1, Bio-rad) or an iBright™ FL1500 Imaging System (Thermo Fisher Scientific).

**Table 3:** List of antibodies used in Western blot analysis

Antibodies (clone)	Antibody dilution	Catalog number (Origin)	Secondary antibody, Catalog number (Origin)
SOX9	1:1,000	#AB5535 (Chemicon, Billerica, MA, USA)	Goat anti-rabbit secondary antibody conjugated to horseradish peroxide (HRP) (#1706515, Bio-Rad)
COMP	1:1,000	#SAB1411896-50 (Sigma)	Goat anti-mouse secondary antibody conjugated to HRP (#1706516, Bio-Rad)
β-ACTIN (4C2)	1:1,000	#MABT825 (Merck Millipore)	Goat anti-mouse secondary antibody conjugated to HRP (#1706516, Bio-Rad)

### Statistical analysis

All experiments were performed using MSCs derived from ten independent patient donors (N = 10 per source). The data were expressed as the mean ± SEM. To quantify multiple differentiation capacities by morphology, the differentiation value (DV) method of the histological and western blot data was adopted [3, 17]. The differentiation value (DV) method was used to semi-quantitatively compare staining intensity and positive area percentages across different MSC sources. This approach allows standardized image-based comparison while minimizing observer bias. However, DV reflects relative staining intensity rather than direct functional bio-mechanical properties. Briefly, five random high-power fields from one source were measured and calculated using ImageJ software (NIH, Bethesda, MD, USA). The differences between values were determined using a one-way analysis of variance (ANOVA), followed by Turkey’s HSD post-hoc test. Statistical analysis was performed using GraphPad Prism 5.0 (GraphPad Software, San Diego, CA, USA). A *p* value was considered statistically significantly different at *p* < 0.05.

## Results

### Morphology of BM-MSCs, AD-MSCs and NP-MSCs

**Fig. 1:**
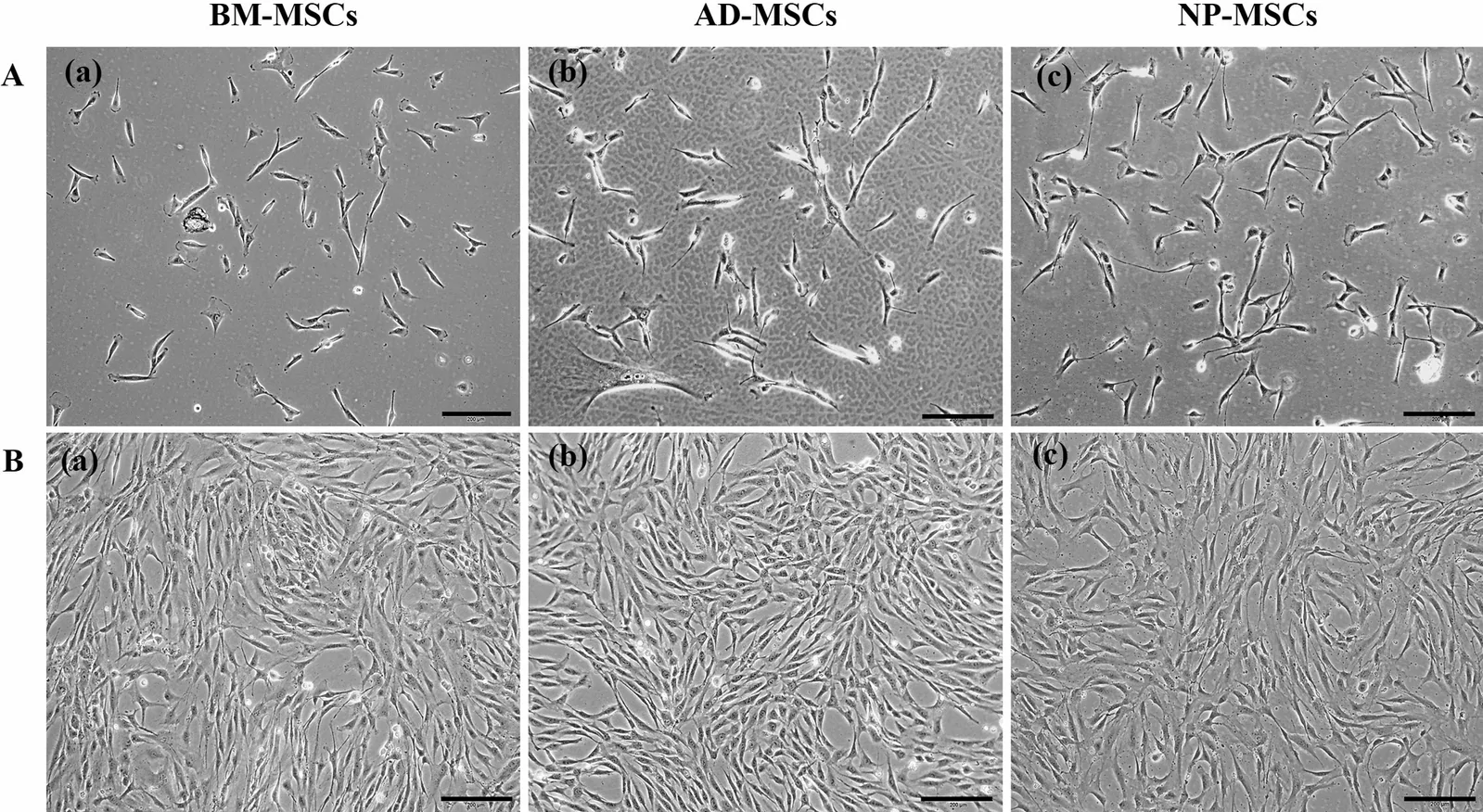
Growth colony and morphology of isolated bone marrow– (BM–MSCs), adipose– (AD–MSCs) and nucleus pulposus– (NP–MSCs) derived mesenchymal stem cells. **A** A typical fibroblast–like morphology of the primary culture (at passage 0) was observed within 7 days of culture of BM–MSCs (a), AD–MSCs (b), and NP–MSCs (c). **B** The representative images at 80% confluences of BM–MSCs (a), AD–MSCs (b), and NP–MSCs (c) at passage 1. (Original magnification = 100 × , bar = 200 μm)

For MSCs, cell lines were established from isolation, expansion to cryopreserved banking cultivation, and expansion reached around 1.0 × 10^7^ cells of BM-MSCs, AD-MSCs, and NP-MSCs, which have the average duration as 34.6 ± 8.1, 19.7 ± 7.3, and 28.8 ± 5.8 days, respectively, and the passage number of BM-MSCs, AD-MSCs, and NP-MSCs is 3.4 ± 0.5, 2.6 ± 0.7, and 3.2 ± 0.8 passages, respectively. Based on phase-contrast microscopic appearance, BM-MSCs, AD-MSCs and NP-MSCs obtained from all ten donors demonstrated a classical fibroblast morphology within 7 days of culture of primary cells at passage 0 (Fig. 1A (a–c)). Then, cells were grown in culture medium until 80% confluence (Fig. 1B (a–c)). After that, cells were harvested to continue expanding and prepared for the experiment and cryopreservation.

### Cell proliferation capacity of BM-MSCs, AD-MSCs and NP-MSCs

**Fig. 2:**
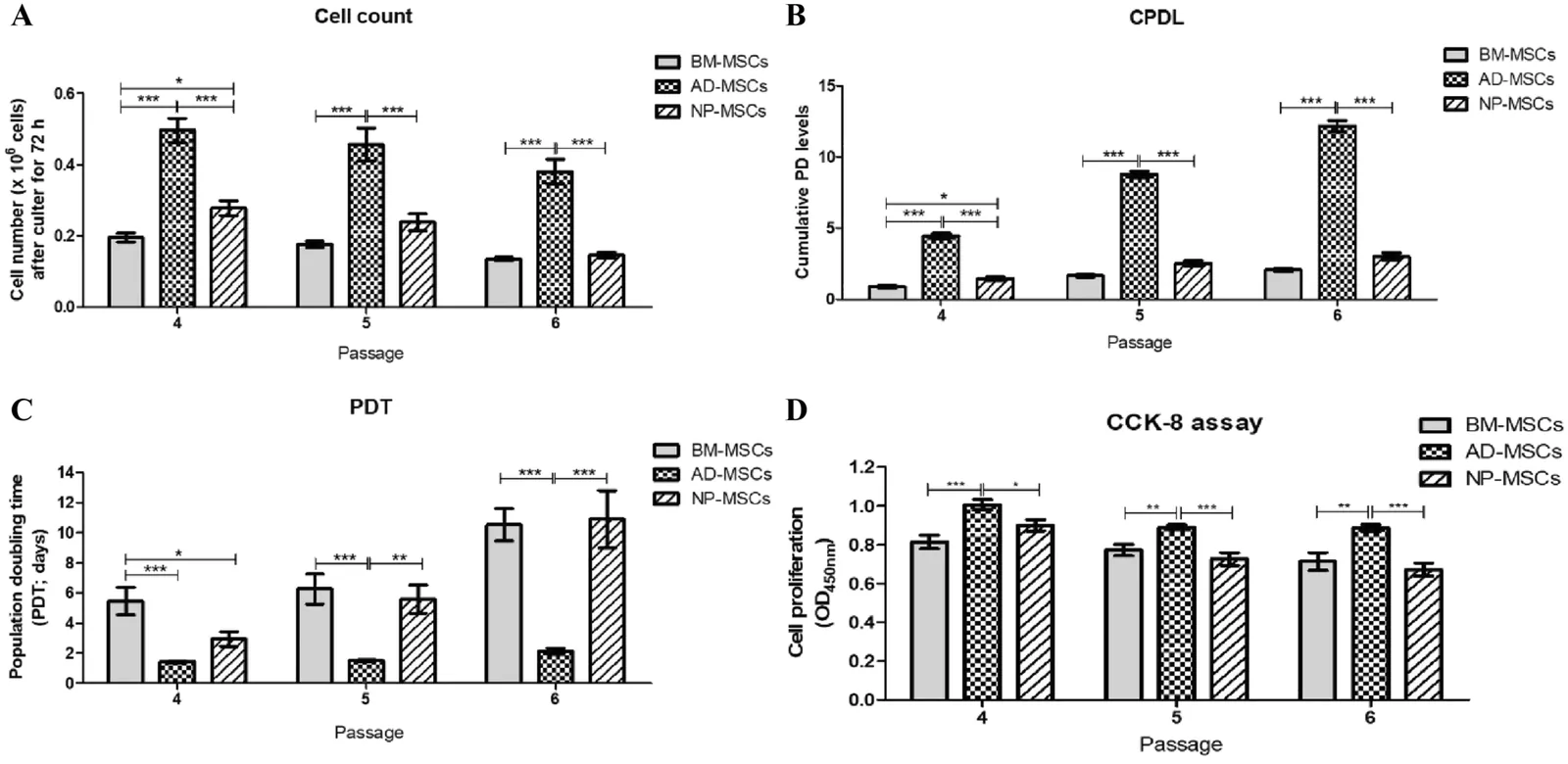
Comparative study of proliferation capacity of bone marrow- (BM-MSCs), adipose- (AD-MSCs), and nucleus pulposus- (NP–MSCs) derived mesenchymal stem cells at passage 4 to passage 6. **A** Cell count number, **B** cumulative population doubling level (CPDL), **C** population doubling time (PDT), and **D** Cell counting kit-8 (CCK-8) assays of BM-MSCs, AD-MSCs and NP-MSCs. All values are shown as means ± standard deviation; *N* = 10/source. (*p < 0.05, **p < 0.01, ***p < 0.001)

We next evaluated the cell proliferation capacity of BM–MSCs, AD–MSCs, and NP–MSCs by calculating cell count numbers with mathematical formulae with previous report [15, 16]. We found that AD–MSCs exhibited a higher proliferation activity than other BM–MSCs and NP–MSCs (*p* < 0.001; Fig. 2A). As shown by cumulative population doubling level (CPDL) and population doubling time (PDT) (all P4-P6 (*p* < 0.001); Fig. 2B, C), plots showed that AD–MSCs possessed higher PD numbers for each passage compared with BM–MSCs and NP–MSCs (all P4-P6 (*p* < 0.001)), indicating that AD–MSCs had greater proliferative potential than BM–MSCs and NP–MSCs (Fig. 2B). At all passages (P4-P6), PDT of AD–MSCs, NP–MSCs, and BM–MSCs reached 2.12 ± 0.5 days, 10.52 ± 0.5 days, and 10.90 ± 0.5 days, respectively (*p* < 0.001; Fig. 2C).

We next confirmed the cell proliferation capacity of BM–MSCs, AD–MSCs, and NP–MSCs by using cell counting kit–8 (CCK–8) assay. AD–MSCs also possessed higher OD values when compared with NP–MSCs and BM–MSCs (all passages (P4-P6) were shown to be different with *p* < 0.05, *p* < 0.01, and *p* < 0.001; Fig. 2D). Based on these experimental findings, AD–MSCs had the highest proliferative potential when compared with other MSCs sources (NP–MSCs and BM–MSCs).

### Immunophenotype of BM-MSCs, AD-MSCs and NP-MSCs

**Fig. 3:**
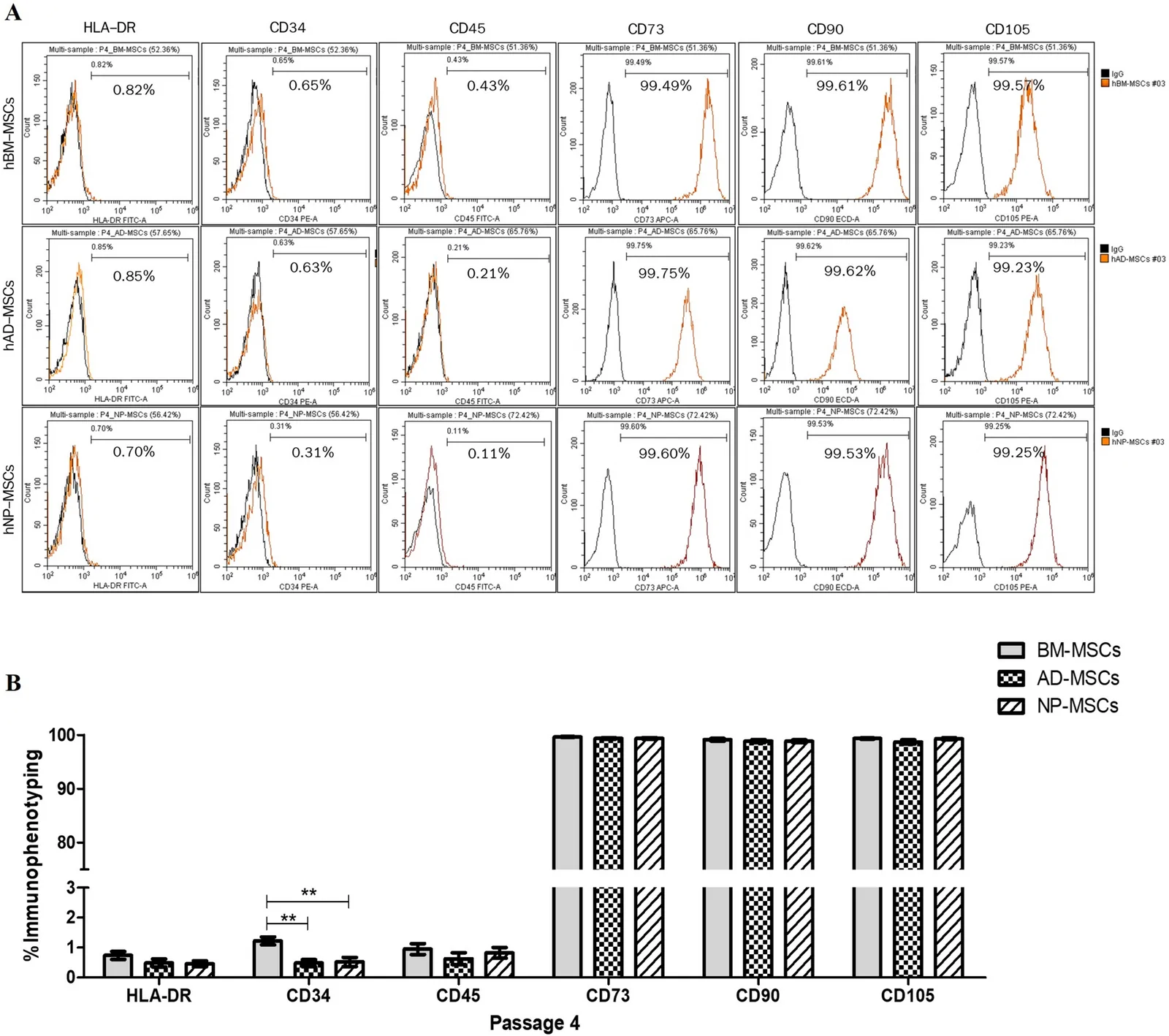
Immunophenotyping of isolated bone marrow– (BM–MSCs), adipose– (AD–MSCs) and nucleus pulposus– (NP–MSCs) derived mesenchymal stem cells. **A** Flow cytometry analysis showed that BM–MSCs, AD–MSCs, and NP–MSCs consistently stained positive for CD73, CD90, and CD105 and did not react with CD34, CD45, and HLA–DR (*N* = 5; one representative example shown). **B** A representative graph of immunophenotyping of BM–MSCs, AD–MSCs, and NP–MSCs. Cell percentage of positive expressing cells with MSC marker (CD73, CD90, and CD105). Cells were negative for hematopoietic markers (HLA–DR, CD34, and CD45). All values are shown as means ± standard deviation; n = 10/source (**p < 0.01)

Based on the MSC properties using the International Society for Cells and Gene Therapy (ISCT) standard criteria for the identification of MSCs with a series of cell surface antigens of MSC immunophenotyping [18] were analyzed by flow cytometry. As shown in Fig. 3A, BM–MSCs, AD–MSCs, and NP–MSCs were positive for the MSC markers, CD73, CD90, and CD105 at high levels (> 95%), and negative for the hematopoietic markers, CD34, CD45, and HLA–DR (< 2%). Representative graphs of BM–MSCs, AD–MSCs, and NP–MSCs showed very similar expression patterns of surface markers (Fig. 3B), but CD34 expression of BM–MSCs was higher than other MSC sources (*p* < 0.01).

### Osteogenic differentiation of BM-MSCs, AD-MSCs and NP-MSCs and their related quantitative assay

**Fig. 4:**
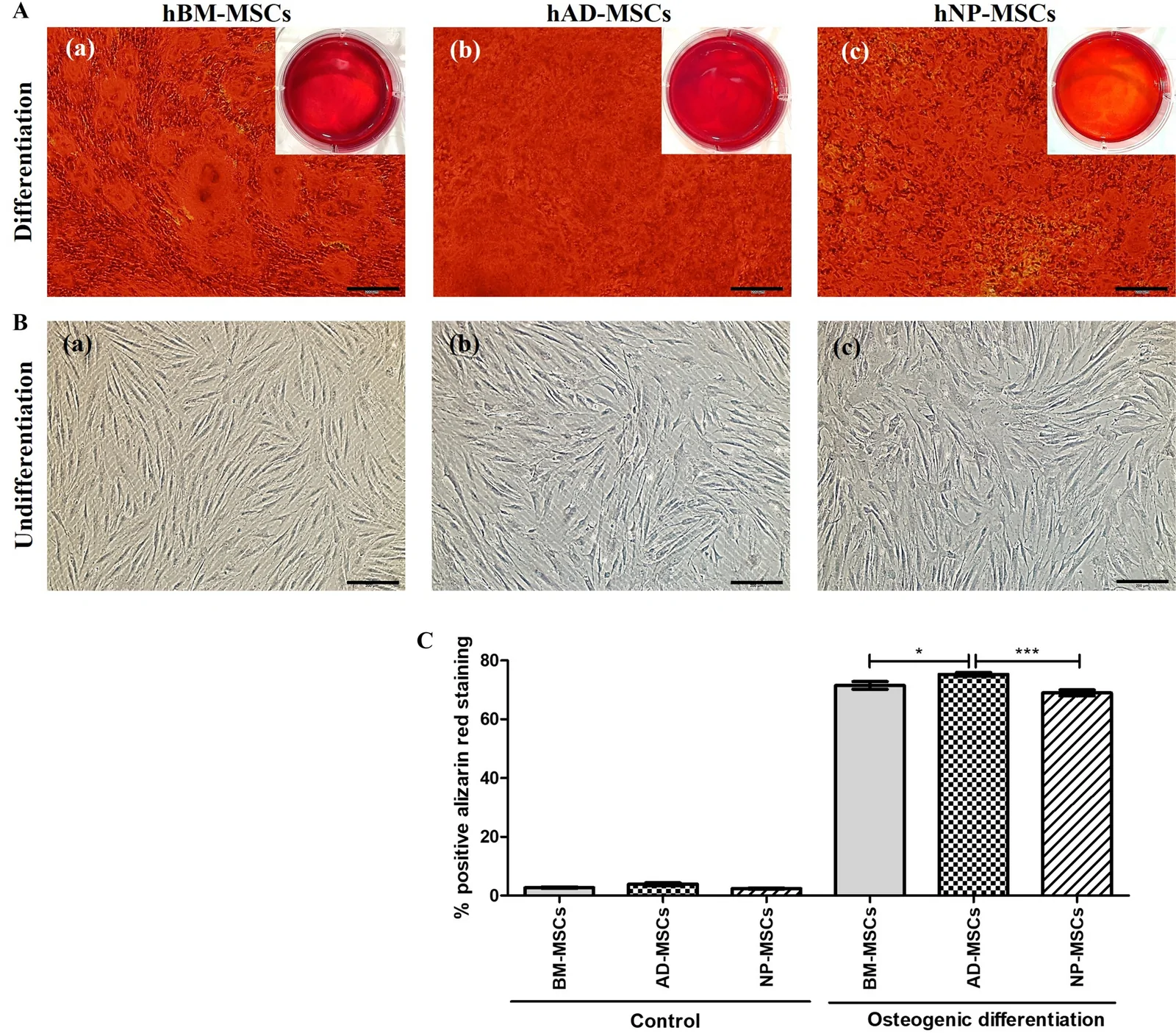
Osteogenic differentiation of bone marrow– (BM–MSCs), adipose– (AD–MSCs) and nucleus pulposus– (NP–MSCs) derived mesenchymal stem cells stained with alizarin red after 4 weeks. **A** BM–MSCs (a), AD–MSCs (b), and NP–MSCs (c) after 4 weeks of osteogenic induction; **B** BM–MSCs (a), AD–MSCs (b), and NP–MSCs (c) undifferentiated control groups. (Original magnification = 100 × , bar = 200 μm). **C** The quantitative analysis of mineral deposition in both cell types, which were cultured in osteogenic medium, showed no difference after 4 weeks. *p < 0.05, ***p < 0.001 versus each control cell. Data represents cells derived from 10 different patients (mean ± SD)

BM–MSCs, AD–MSCs, and NP–MSCs were induced toward osteoblasts to confirm their capacity for MSC differentiation. Calcium deposit was visualized by Alizarin Red in all hMSCs sources (Fig. 3A (a–c)) after 4 weeks of induction, and negative mineralized matrix in all undifferentiated hMSCs (Fig. 4B (a–c)). As shown in Fig. 4C, the area percentage showed that AD–MSCs had superior osteogenic differentiation capacity at 75.31 ± 9.20% after 4 weeks of induction when compared to BM–MSCs (*p* < 0.05) and NP–MSCs (*p* < 0.001), which were 71.50 ± 4.39% and 68.99 ± 7.34%, respectively.

### Adipogenic differentiation of BM-MSCs, AD-MSCs and NP-MSCs and their related quantitative assay

**Fig. 5:**
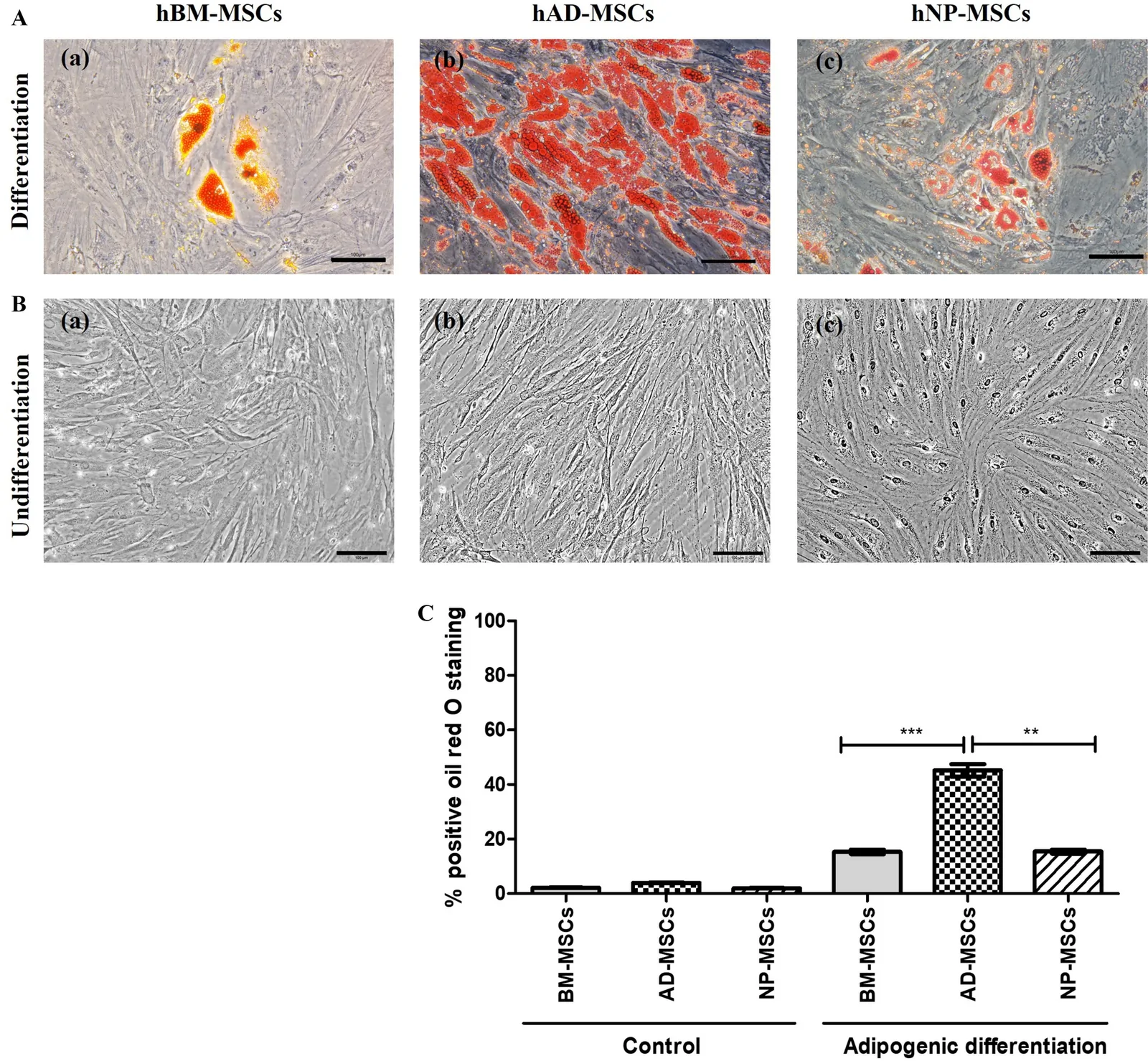
Adipogenic differentiation of bone marrow– (BM–MSCs), adipose– (AD–MSCs) and nucleus pulposus– (NP–MSCs) derived mesenchymal stem cells stained with alizarin red after 3 weeks. **A** BM–MSCs (a), AD–MSCs (b), and NP–MSCs (c) after 3 weeks of adipogenic induction; **B** BM–MSCs (a), AD–MSCs (b), and NP–MSCs (c) undifferentiated control groups. (Original magnification = 200 × , bar = 100 μm). **C** Quantitative analysis of accumulated neutral lipid deposition in both cell types cultured in adipogenic medium showed no difference after 3 weeks. **p < 0.01, ***p < 0.001 versus each control cell. Data represents cells derived from 10 different patients (mean ± SD)

Adipogenic differentiation of BM–MSCs, AD–MSCs, and NP–MSCs was confirmed by oil red O staining after 3 weeks of induction. Lipid droplet accumulation in all hMSCs sources was visualized by oil red O at different levels (Fig. 5A (a–c)), and negative accumulated oil droplets in all undifferentiated hMSCs (Fig. 5B (a–c)). As shown in Fig. 4C, the area percentage showed that AD–MSCs had superior adipogenic differentiation capacity, as it was 45.18 ± 3.41% after 3 weeks of induction when compared to BM–MSCs (*p* < 0.001) and NP–MSCs (*p* < 0.01), which were 15.30 ± 3.41% and 15.43 ± 3.41%, respectively.

### Chondrogenic differentiation of BM-MSCs, AD-MSCs and NP-MSCs and their related quantitative assay

**Fig. 6:**
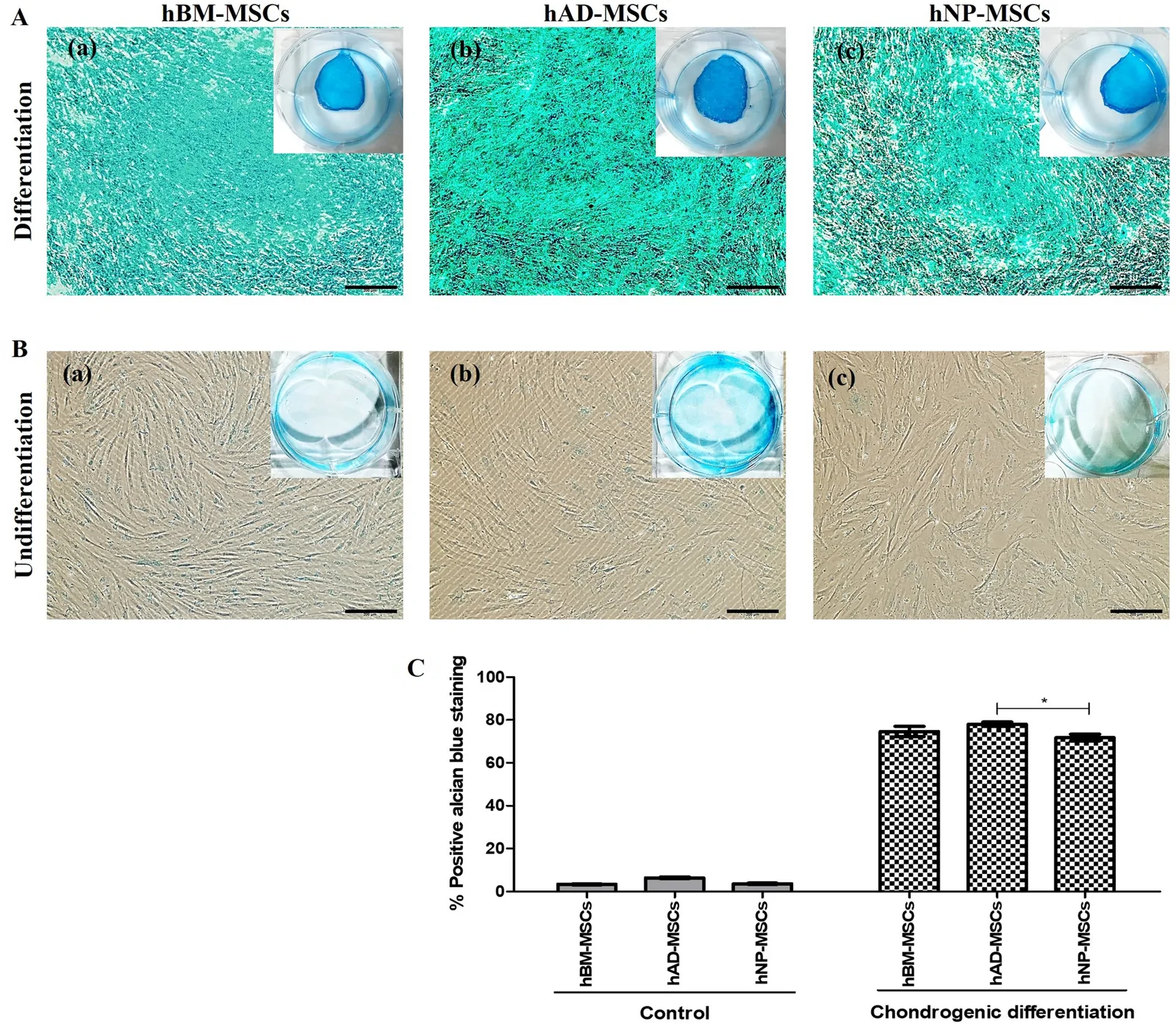
Chondrogenic differentiation of bone marrow– (BM–MSCs), adipose– (AD–MSCs) and nucleus pulposus– (NP–MSCs) derived mesenchymal stem cells stained with alcian blue after 3 weeks. **A** BM–MSCs (a), AD–MSCs (b), and NP–MSCs (c) after 3 weeks of adipogenic induction; **B** BM–MSCs (a), AD–MSCs (b), and NP–MSCs (c) undifferentiated control groups. (Original magnification = 100 × , bar = 200 μm). **C** Quantitative analysis of sulfated proteoglycans deposition in both cell types cultured in chondrogenic medium showed no difference after 3 weeks. *p < 0.05 versus each control cell. Data represents cells derived from 10 different patients (mean ± SD)

Chondrogenic differentiation of BM–MSCs, AD–MSCs, and NP–MSCs was confirmed by Alcian blue staining after 3 weeks of induction. Sulfated glycosaminoglycan deposition was observed in all hMSC sources, with varying intensities (Fig. 6A (a–c)), whereas no sulfated proteoglycan deposition was detected in undifferentiated hMSCs (Fig. 6B (a–c)). Quantitative analysis (Fig. 6C) showed that AD–MSCs exhibited the highest mean area percentage (78.02 ± 3.41%), followed by BM–MSCs (74.62 ± 3.41%) and NP–MSCs (71.81 ± 3.41%). A statistically significant difference was observed between AD–MSCs and NP–MSCs (*p* < 0.05), while no significant difference was found between AD–MSCs and BM–MSCs. Although AD–MSCs tended to show greater chondrogenic differentiation based on staining intensity, this trend was not consistently statistically significant across all comparisons.

### RT-qPCR analysis of chondrocyte-specific marker expression

**Fig. 7:**
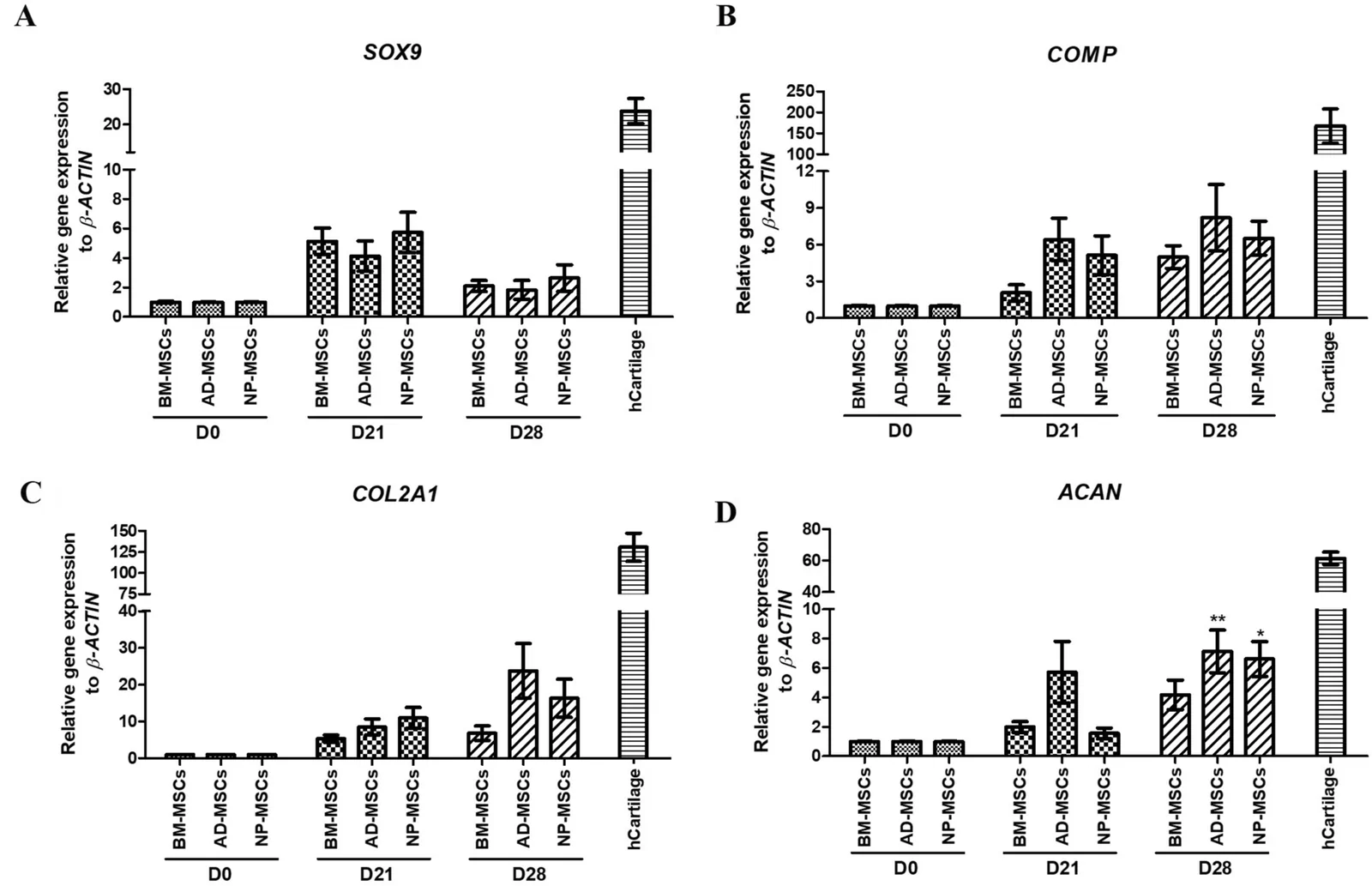
RT–qPCR analysis of chondrogenic differentiation of bone marrow– (BM–MSCs), adipose– (AD–MSCs) and nucleus pulposus– (NP–MSCs) derived mesenchymal stem cells after 3–4 weeks. **A** *SOX9*, **B** *COMP*, **C** *COL2A1*, and **D** *ACAN*. Gene expression was normalized to corresponding *β-ACTIN* and calculated by relative expression compared to each control cell. *p < 0.05, **p < 0.01 versus each control cell. Data represents cells derived from 10 different patients (mean ± SD)

To evaluate chondrogenic differentiation–related genes (*SOX9, COMP, Col2A1,* and *ACAN*) were examined by RT–qPCR analysis on days 21 and 28 of induction (Fig. 7A–D). *SOX9* transcription factor was detected with higher mRNA expression on day 21 in all hMSCs, followed by a decrease on day 28 of induction. The mRNA expressions of *COMP, Col2A1,* and *ACAN* were also detected on day 21 in all hMSCs, followed by an increase on day 28 of induction.

### Western blot analysis of chondrocyte-specific marker expression

**Fig. 8:**
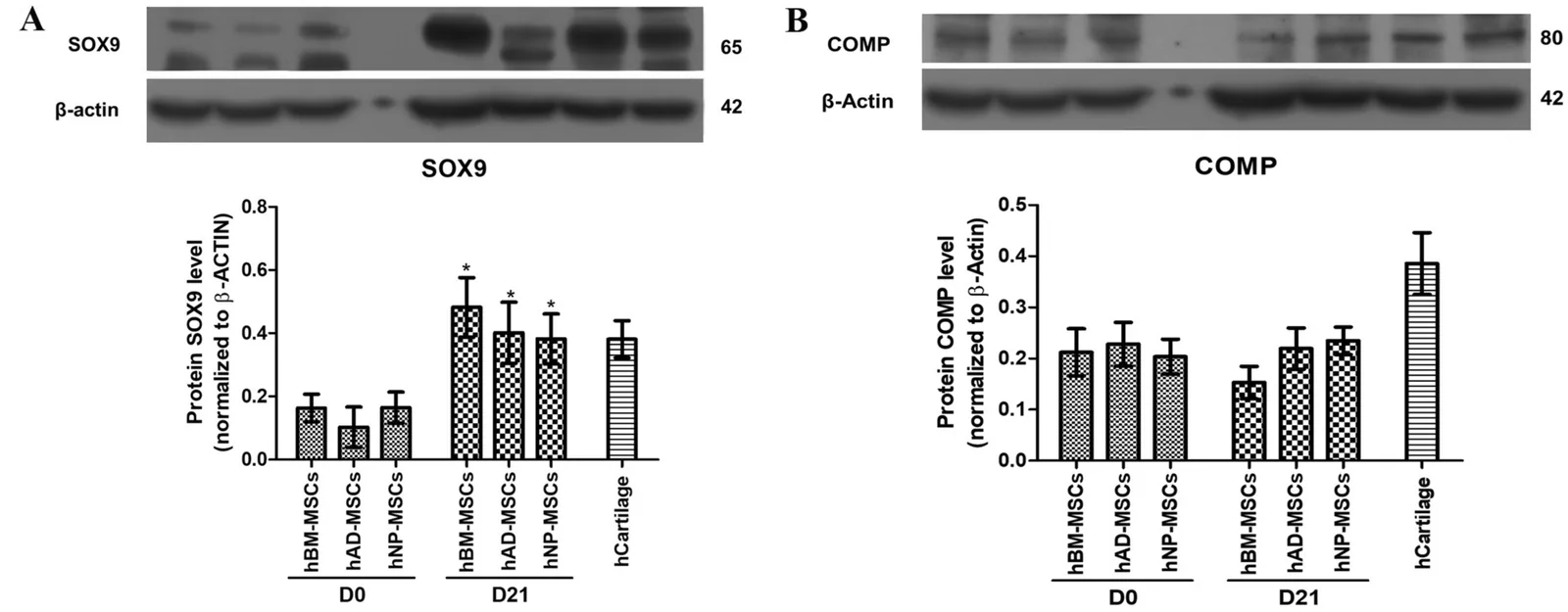
Semi–quantitative Western blot analysis of chondrogenic differentiation of bone marrow– (BM–MSCs), adipose– (AD–MSCs) and nucleus pulposus– (NP–MSCs) derived mesenchymal stem cells after 3 weeks. **A** SOX9 and **B** COMP. β-actin was used as an internal control. *p < 0.05 versus each control cell and between different D21 groups. Data represents cells derived from 10 different patients (mean ± SD)

To further confirm the mRNA expression of the chondrocyte-specific markers after 21 days of induction. Western blot analysis was performed, and the expressions of SOX9 and COMP (Fig. 8A–B) were detected at a similar level in all hMSCs of chondrocyte differentiation. The expression of β-actin was used as a housekeeping gene and protein control. The protein expression of these genes detected by Western blot analysis was very similar to mRNA expression by RT-qPCR analysis. These results indicated that all hMSCs can induce chondrogenic differentiation at a similar level of gene and protein expressions.

## Discussion

Degenerative intervertebral disc disease (DIVDD) remains a major contributor to chronic low back pain worldwide. Current treatment strategies, including physical therapy, analgesics, and surgical interventions, primarily address symptoms but fail to restore the native disc structure or function. Consequently, regenerative approaches, particularly those utilizing mesenchymal stem cells (MSCs), have gained increasing attention for their potential to restore extracellular matrix (ECM) and cellularity within the nucleus pulposus (NP) [1]. In the present study, we compared MSCs derived from three tissue sources-bone marrow (BM-MSCs), adipose tissue (AD-MSCs), and nucleus pulposus (NP-MSCs) harvested from the same patients with DIVDD. This design minimizes inter-patient variability and provides a robust intra-individual comparison of proliferation capacity, immunophenotype, and multipotent differentiation ability. Our findings demonstrated that AD-MSCs possessed superior proliferative capacity compared with BM-MSCs and NP-MSCs, while all three sources exhibited similar chondrogenic differentiation potential at both gene and protein expression levels. However, the expression of these markers (SOX9 and COMP) serves as a proxy and does not definitively guarantee the synthesis of a biomechanically competent tissue under physiological conditions.

A key strength of this study is its paired design, in which stem cells were isolated from multiple tissue sources within the same patient. This approach minimizes donor-related confounding factors, including age, comorbidities, and genetic variability, which often limit cross-sectional comparisons [2]. In addition, a comprehensive evaluation framework was employed, encompassing morphological assessment, cell surface marker characterization, tri-lineage differentiation assays (adipogenic, osteogenic, and chondrogenic), and molecular analyses using RT-qPCR and Western blot. This multi-level validation strengthens the reliability of the observed differences among MSC sources.

Our findings further identify adipose tissue as a particularly advantageous source of MSCs. Adipose tissue harvesting is minimally invasive compared with bone marrow aspiration and is associated with lower donor site morbidity. Moreover, the higher proliferative capacity observed in AD–MSCs suggests a practical advantage for large-scale expansion and potential clinical application [9]. While BM–MSCs have traditionally been considered the “gold standard” for musculoskeletal regeneration [5], their yield and functional capacity are known to decline with advancing donor age, which may limit their use in older populations where degenerative intervertebral disc disease is most prevalent [19]. Consistent with this, BM–MSCs in our study demonstrated relatively slower proliferation compared with AD–MSCs.

AD–MSCs exhibited a higher proliferative capacity than both BM–MSCs and NP–MSCs, in agreement with previous studies reporting superior expansion potential of adipose-derived MSCs [2, 6]. This enhanced proliferative ability may be related to intrinsic biological differences between tissue sources. Prior studies have suggested that AD–MSCs display greater metabolic flexibility, increased resistance to oxidative stress, and a more active paracrine signaling profile compared with BM–MSCs [6, 9]. These features may confer a survival and expansion advantage, particularly under nutrient-limited or hypoxic conditions resembling the intervertebral disc microenvironment. However, these underlying mechanisms were not directly examined in the present study and warrant further investigation.

Previous comparative studies have reported conflicting results regarding the chondrogenic potential of MSCs from different sources. Dai et al. demonstrated that AD-MSCs differentiated into NP-like cells with similar efficiency to BM-MSCs when cultured in three-dimensional systems [2]. Similarly, Li et al. reported that NP-MSCs retain multipotency but may exhibit reduced regenerative potential in advanced degeneration [3]. Our findings support these observations, showing that while AD-MSCs excel in proliferation and adipogenesis, the chondrogenic capacity of BM-MSCs, AD-MSCs, and NP-MSCs was broadly similar at both transcriptional and protein levels. It is notable that NP-MSCs, despite being tissue-specific, showed limited proliferation compared with AD-MSCs. This limitation aligns with prior reports indicating that NP-derived stem cells are susceptible to the hypoxic, acidic, and nutritionally deprived microenvironment characteristic of degenerative discs [7]. Nevertheless, their phenotypic similarity to NP cells suggests that, with appropriate optimization strategies, NP-MSCs may still hold promise for disc-specific repair.

Our results suggest that AD-MSCs offer practical advantages in terms of accessibility and expansion capacity. However, because chondrogenic differentiation was comparable among all three MSC sources at gene and protein levels, these findings do not establish functional superiority in disc regeneration. Rather, AD-MSCs may represent a feasible and scalable option for clinical translation, pending further validation under disc-mimicking and *in vivo* conditions. Adipose tissue is abundant, collection is minimally invasive, and cells can be rapidly expanded to clinically relevant numbers. These properties render AD-MSCs attractive candidates for the development of autologous or allogeneic stem cell banks that may facilitate timely regenerative interventions. Given that BM-MSCs and NP-MSCs showed comparable chondrogenic differentiation to AD-MSCs, they should not be dismissed; rather, the choice of source may depend on the clinical context. For example, AD-MSCs may be preferred for rapid large-scale expansion, whereas NP-MSCs might be combined with biomaterials or signaling factors to enhance disc-specific regeneration.

An additional limitation relates to the use of standard chondrogenic induction media under normoxic monolayer culture conditions. These experimental settings may favor classical MSC chondrogenesis but do not fully replicate the hypoxic, acidic, hyperosmotic, and mechanically loaded microenvironment of the native intervertebral disc. It is possible that disc-mimicking conditions could differentially influence MSC survival, matrix synthesis, and phenotype stability, particularly for NP-derived MSCs that are adapted to this niche. Therefore, the present findings should be interpreted within the context of conventional chondrogenic assays, which may not fully predict *in vivo* regenerative performance. The primary limitation of this study is its *in vitro* design, which cannot fully replicate the harsh microenvironment of the degenerated intervertebral disc. Factors such as low oxygen tension, high osmotic pressure, and mechanical loading are known to significantly affect MSC survival and differentiation [7]. Furthermore, our sample size was relatively small (10 patients), all donors were older adults (mean 63.7 years) with advanced degeneration (Pfirrmann grade III–V degeneration). As a result, the functional capacity of NP-MSCs may be underestimated, as they were harvested from a biologically compromised microenvironment. These findings may therefore differ in younger patients, where a more favorable niche could better preserve their regenerative potential. Finally, while we demonstrated chondrogenic gene and protein expression, functional properties of the newly synthesized ECM (e.g., proteoglycan content, biomechanical stiffness) were not evaluated. Future studies should expand upon these findings by incorporating functional extracellular matrix assays to assess the quality and biomechanical competence of differentiated cells. *In vivo* validation using animal models of disc degeneration is critical to determine whether the observed chondrogenesis translates to durable structural repair and symptom relief. Moreover, optimization of NP-MSCs through the use of growth factors (e.g., TGF-β3, Wnt pathway modulators) or biomaterial scaffolds may enhance their regenerative potential [20]. Comparative trials in younger versus older donors would also clarify the impact of age on MSC potency.

Nonetheless, limitations should be acknowledged. All experiments were conducted *in vitro*, without *in vivo* validation of functional disc regeneration. Donor variability (age, Pfirrmann grade) may also have influenced MSC characteristics. Future work should incorporate *in vivo* studies, assess MSC–ECM interactions under disc-mimicking conditions, and explore molecular strategies to optimize chondrogenic induction.

In summary, while BM-, AD-, and NP-MSCs share core stemness features AD-MSCs demonstrated superior proliferative capacity and ease of harvest, while chondrogenic differentiation capacity was broadly comparable among BM-, AD-, and NP-MSCs. Therefore, AD-MSCs may offer logistical advantages for translational applications; however, functional superiority in disc regeneration remains to be established. BM-, AD-, and NP-MSCs have similar biological advantages in multilineage differentiation potential (osteoblasts, chondrocytes, and adipocytes), especially chondrogenic differentiation potential was greater expressed at both gene and protein levels; these biological advantages should be considered systematically when choosing the MSC source for a specific clinical application, especially cartilage and/or IVD tissue engineering for the treatment of DIVDD.

## Data Availability

The data that supports the findings in this study are available from the corresponding authors upon reasonable request.
